# Adapted Fencing for Patients With Invasive Breast Cancer: The RIPOSTE Pilot Randomized Controlled Trial

**DOI:** 10.3389/fspor.2022.786852

**Published:** 2022-03-29

**Authors:** Abdou Y. Omorou, Didier Peiffert, Christine Rotonda, Aurélie Van Hoye, Edem Allado, Oriane Hily, Margaux Temperelli, Bruno Chenuel, Dominique Hornus-Dragne, Mathias Poussel

**Affiliations:** ^1^Université de Lorraine, CHRU-Nancy, Inserm CIC-1433 Clinical Epidemiology, Nancy, France; ^2^Université de Lorraine, Apemac, Nancy, France; ^3^The French National Platform Quality of Life and Cancer, Nancy, France; ^4^Lorraine Institute of Oncology, Department of Radiation Oncology, Vandoeuvre-Lès-Nancy, France; ^5^Université de Lorraine, Centre Pierre Janet, Metz, France; ^6^Université de Lorraine, CHRU-Nancy, University Centre of Sports Medicine and Adapted Physical Activity, Nancy, France; ^7^Université de Lorraine, DevAH, Department of Physiology, Nancy, France; ^8^Médipole Garonne, Department of Anaesthesiology, Toulouse, France

**Keywords:** adapted physical activity, fencing, breast cancer, quality of life, clinical trial

## Abstract

**Introduction:**

Even if indications for mastectomy have been progressively reduced in loco-regional breast cancer (BC) treatment, the harmful effects of surgery are still numerous and can impact physical and psychological wellbeing of women. The RIPOSTE (Reconstruction, self-Image, Posture, Oncology, “Santé”-Health, Therapy, “Escrime”-Fencing) program aimed to propose adapted fencing to patients with BC. This study aims to investigate the effect and conditions of effectiveness of the RIPOSTE program.

**Methods and analysis:**

This is a prospective randomized controlled trial including 24 patients with invasive BC who have just undergone surgery. The study will be proposed to the patient and if interested, the patient will be referred to a sports physician for a medico-sportive evaluation. At the end the evaluation, if the patient meets the inclusion criteria, she will be randomly assigned to one of the 2 groups based on a 1:1 principle: *Early RIPOSTE group* (receive one fencing session per week for 3 months immediately after their inclusion), *Delayed RIPOSTE group* (receive one fencing session per week for 3 months but within the 3 months following their inclusion). Patients will be included for 6 months with 3 follow-up times (0, 3, and 6 months) by a sport physician. The primary outcome is the evolution of quality of life score. Secondary outcomes are disability score, fatigue, anxiety-depression, cost-effectiveness and process evaluation.

**Ethics and dissemination:**

The study protocol has been approved by a French ethics committee (CPP Sud Méditerranée IV, N°ID-RCB: 2020-A01916-33). Results will be submitted for publication, at scientific conferences and through press releases.

**Trial Registration:**

NCT04627714.

## Introduction

Despite national cancer screening programme organized since 2004, breast cancer (BC) still affects many women in France (Jooste et al., 2011). Recent therapeutic improvements have significantly improved the survival rate of patients (89% of 5-year survival rate). Surgery is the essential treatment for BC at the loco-regional stage. Even though the indications for mastectomy have been gradually reduced and conservative surgery is more frequently possible, the harmful effects of surgery are still numerous (pain, mobility problems, oedema…). Surgical scars can hamper the mobility of the arm and posture is very often changed after mastectomy (for example, the shoulder may lower and become internally rotated and back pain is also frequently found) (Rostkowska et al., 2006). Lymph node expertise by sentinel procedure as well as lymph node axillary dissection are also frequently reported as possible causes of postoperative pain and joint amplitude reduction. In addition to surgery, adjuvant treatments are also involved in numerous adverse effects enhancing pain. For instance, radiotherapy may increase the risk of lymphoedema and the use of aromatase inhibitor hormone therapy has been associated with possible risk of arthralgia.

Many studies have shown that the side-effects of the various treatments (e.g., pain in the treated area, redness of the skin, oedema of the breast, nausea, fatigue…) lead to a deterioration in the Quality of Life (QoL) of patients (Browall et al., 2008; Montazeri, 2008). This mainly concerned the areas of physical functions, daily activities, and body image. An increase in symptoms (fatigue, dyspnoea, pain, nausea/vomiting and constipation) as well as high anxiety/depression were also observed (Reich et al., 2008).

Literature have shown benefits of regular physical activity (PA) during and after BC treatment, particularly in terms of improving the quality of life (QoL) (Manneville et al., 2018). Several randomized controlled trials have shown that appropriate physical activity (PA) during and after cancer treatment improved patients' QoL (Mishra et al., 2012a,b). However, further studies are needed to determine the optimal type and timing of exercise (Desnoyers et al., 2016).

With 119 Olympic medals since the modern Olympics began in 1896, fencing is France's number one Olympic sport. The relevance of fencing as PA in the context of BC has been suggested quite naturally (both by patients and the medical profession) during the various consultations in the management of this pathology. The aim of fencing is to hit the opponent and avoid being hit back. Its practice thus makes it possible to acquire (or to maintain) the desire to fight and to win. Created in 2014 by Dr. Dominique Hornus-Dragne (MD, Toulouse, France), the RIPOSTE (Reconstruction, self-Image, Posture, Oncology, “Santé”-Health, Therapy, “Escrime”-Fencing) program aimed to propose adapted fencing to patients with BC (Leroy, 2017; Meyer et al., 2017). The program is under the governance of a scientific committee responsible for the deliverance of the RIPOSTE Label. To obtain this RIPOSTE Label, professional fencing masters have to complete a specific course of 3 days, renewed every 2 years, and to adhere to a 10 points chart (dedicated fencing clothing and material, free of charge for patients, under medical supervision). Adapted fencing is performed using the saber, always held on the operated side (sometimes involving change in laterality). The choice of the saber is important because this weapon is light and allows a large range of motion of the injured side during attacks, parries and ripostes.

An evaluation of the effectiveness of this programme and the most opportune period for its implementation is necessary to justify its maintenance and extension on a national and international scale. Before setting up a large-scale study, a pilot study on the expected effect and conditions of effectiveness of the RIPOSTE program is necessary.

### Research Hypotheses and Objectives

#### Hypotheses

Adapted and secure fencing and its' early implementation in immediate poste-surgery may be an effective way of improving or reducing deterioration of physical and psychological health in patients operated for BC.

#### Main Objective

The main objective is to compare immediate post-surgical fencing (ERG: Early RIPOSTE group) vs. delayed fencing (DRG: Delayed RIPOSTE group) on 6-month evolution of global QoL score of patients operated for invasive BC.

#### Secondary Objectives

- (SO1) Compare ERG vs. DRG on the 6-month evolution of several secondary criteria of patients operated for invasive BC: other dimensions of QoL, disability of the lateral arm, fatigue and anxiety-depression.
- (SO2) Compare early (3-months) effectiveness of fencing (fencing vs. no fencing) on physical and psychological health of patients operated for BC.
- (SO3) Identify 6-month trajectories of QoL, disability of lateral arm, fatigue and anxiety-depression and theirs associated factors.
- (OS4) Evaluate retrospectively the conditions of effectiveness of RIPOSTE (qualitative interview).

## Methods and Analysis

### Design of RIPOSTE Trial

A prospective randomized controlled trial including patients with invasive BC who have just undergone surgery from 3 centers in Northeastern France (CHRU Nancy, Institut Lorrain de Canérologie and HIA Legouest Metz). The study will be proposed to the patient by the oncologist or surgeon after surgery. If interested, the patient will be referred to a sports physician for a medico-sportive evaluation.

#### Inclusion Criteria

- A woman aged at least 18 years;
- Undergone unilateral breast surgery for primary invasive breast cancer;
- 2–4 weeks after surgery;
- Accept and be able to complete the self-administered questionnaires;
- Satisfy the prior medico-sportive evaluation;
- Informing and signing informed consent.

#### Exclusion Criteria

- Presence of bone metastases, especially vertebral metastases;
- Presence of a contraindication to the practice of fencing at the time of the medico-sport evaluation;
- Refusal to participate in the study.

### Recruitment and Randomization

The study is to be initiated from September 2021 with an anticipated end for subject recruitment in November 2021. Study completion is scheduled for June 2022.

**Figure 1:**
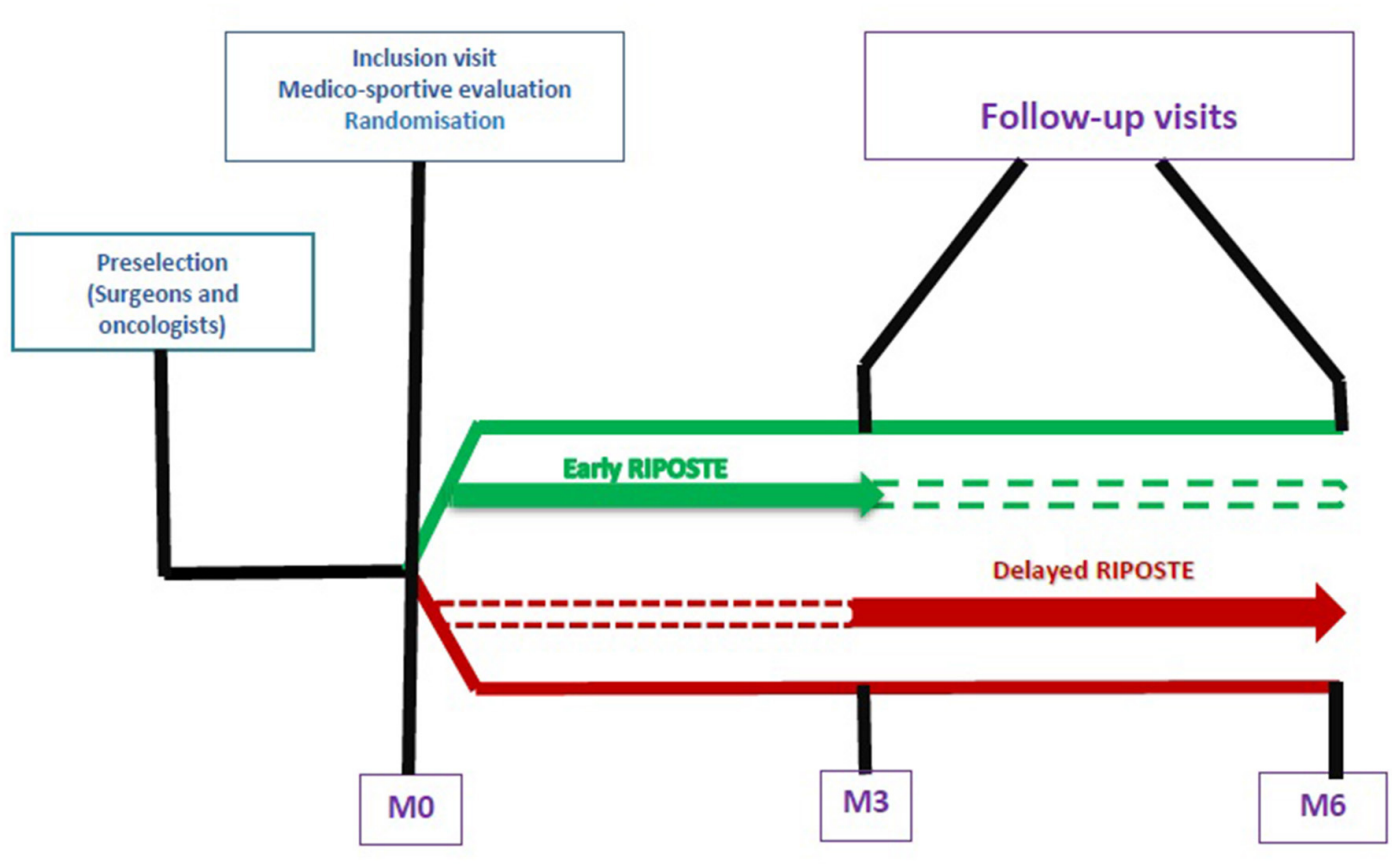
Design of the ≪Solution RIPOSTE≫ trial.

The study will be proposed to the patient by the oncologist or surgeon after surgery. If interested, the patient will be referred to a sports physician for a medico-sportive evaluation. At the end the evaluation, if the patient meets the inclusion criteria, she will be randomly assigned to one of the 2 groups based on a 1:1 principle using automatic software Ennov^®^ (Figure 1):

#### Early RIPOSTE Group

Patients in this group will receive one fencing session (1–1.5 h/session) per week for 3 months immediately (within 2 week) after their inclusion. A maximum of 12 sessions.

#### Delayed RIPOSTE Group

Patients in this group will receive one fencing session (1–1.5 h/session) per week for 3 months but within the 3 months following their inclusion. A maximum of 12 sessions.

### Interventions

Once a week, for a duration of 1–1.5 h/session, and during a complete sport season, a group of 10–12 patients practiced adapted fencing. Following a warm up time dedicated to muscle awakening and progressive cardio-respiratory solicitation, patients then started specific fencing exercises from fundamental footwork (advance, retreat, lunge) to actions involving the injured side that holds the saber. Fencing is adapted in so far as all offensive and defensive actions are performed with respect of the mobility of the shoulder. Primary (tierce = outside; quarte = inside; quinte = head) and secondary parries are therefore executed by patients with the highest mobility but without ever causing pain. Coordination, agility and strength are trained during exercises between patients and during lessons with the fencing master. The adapted fencing session finished with a cool down period and a final salute.

### Ethical Approval

Verbal informed consent will be obtained from each participant and they will be allowed to withdraw at any time without any consequences. Data will be made anonymous and only the first letter of the first name and surname and a number attributed to each patient will be recorded. This research complies with the Helsinki declaration and is registered and received ethical authorization by the French committee for individual protection (CPP Sud Méditerranée IV, N°ID-RCB: 2020-A01916-33). This research is registered on clinicaltrials.gov (NCT04627714).

### Follow-Up Visits

Patients will be included for 6 months with 3 follow-up times (0, 3, and 6 months) by a sport physician.

#### Inclusion

Prior to any examination or act specific to the research, the sports physician will inform the patient and obtain her free, informed and written consent. During this visit, after obtaining the patient's written consent, a medico-sportive evaluation will be carried out for each patient by a sports physician to assess her physical conditions for the practice of fencing (assessment of shoulder mobility using the Constant index and evaluation of surgical adhesions) and to detect any contraindications to the practice of fencing. If the medico-sportive evaluation is not satisfactory or if any other inclusion or non-inclusion criteria are not met, the patient will not be included in the study. If all inclusion and non-inclusion criteria are met, the sports physician will proceed with randomization. At the end of this visit, a CRF will be given to the patient to complete the various inclusion questionnaires. She will be offered an appointment for the visit at 3 months regardless of the group in which she is randomized.

#### Month 3 Visit

For patients of the DRG, it will confirm the absence of contraindications to the practice of fencing before the beginning of the sessions within 8 days. At the end of this visit, medical data such as complementary treatments (pain treatment, anti-diarrhea, antiemetic's, psychotropic drugs, treatment not declared to the doctor), weight gain, presence of lymphedema, co-morbidities and patient-reported outcomes will be collected for all patients whatever the intervention group. An appointment for the visit at 6 months will be offered to each patient regardless of the group.

#### Month 6 Visit

This last visit will be devoted to the collection of follow-up data at 6 months (medico-sportive evaluation and administration of questionnaires). If patients do not attend this follow-up visit, a de-identified questionnaire will be sent to the patients' homes within 2 weeks in a pre-stamped, stamped envelope addressed to CIC-1433 EC.

A random selection of 10 patients, 5 from each intervention group, will be contacted by phone to obtain their agreement to participate to the qualitative investigation. An appointment will be made for a retrospective narrative interview at their convenience. It will enable the participants to question their experiences, difficulties and levers, as well as the adaptations made to the programme, in order to identify the indicators of effectiveness and the adaptations necessary for adherence and greater effectiveness of the programme. This interview will be carried out face-to-face by a researcher from the project team.

Two semi-structured interviews of the 2 fencing master will be carried in order to assess the effectiveness indicators, in particular the acceptability of the patients (profiles remaining committed), the difficulties encountered (technical, social, psychological, etc.), the levers (posture, resources, support) and the necessary adaptations (pedagogy, gestures, technique) for better commitment and effectiveness.

### Data Collection

During the study, data on socio-demographic medical and patient-reported outcomes will be collected at each follow-up.

#### Socio-Demographic

Age, marital status, family situation, number of children and their ages, level of education, current professional situation (active or not), socio-professional category, social support and life habits (sleep problems, practice of a physical activity other than fencing,…).

#### Medical

Date of diagnosis of breast cancer, stage of the disease, type of surgery (including lymph node expertise by sentinel procedure or lymph node axillary dissection), type of adjuvant treatment, complementary treatments (pain treatment, anti-diarrhea, antiemetic's, psychotropic drugs, treatment not declared to the doctor), weight gain, presence of lymphedema, co-morbidities.

#### Patient Reported Outcomes

##### Quality of Life

Quality of life will be measured using the EORTC QLQ-C30 and BR23 module. The EORTC (Quality of Life Questionnaire Core 30) questionnaire (“European Organization for Research and Treatment of Cancer”) is a questionnaire developed by Aaronson et al. (1993). It is composed of 30 items and is intended for all patients with cancer, regardless of location. Its psychometric properties were verified in the original version. Patients should answer these questions by circling a number between 1 (“not at all”) and 4 (“a lot”) that best describes their situation at the time of administering the questionnaire. It results in 15 scores of QoL dimensions: physical activities, daily activities, cognitive functions, emotional wellbeing, fatigue, pain, nausea and vomiting, loss of appetite, constipation, diarrhea, financial impact and global health. The QLQ-BR23 questionnaire consists of 23 items to evaluate the symptoms of BC and the side effects of the treatments (Sprangers et al., 1996). It explores 8 different dimensions (4 functional dimensions: body image, sexual activity, sexual pleasure, future perspective and 4 dimensions of symptoms: side effects of treatments, symptoms in the breast, symptoms in the arm and feelings related to the loss of hair). All scores for domains and unique items are linearly transformed from 0 to 100. A high score for the functional domains will express a good functional level, a high score for the general health domain and QoL will translate a good QoL but a high score for areas of symptoms will represent a high level of symptoms. Non-responses are taken into account by assigning them the mean value of the dimension if the number of items to which a response is given is greater than half the items in the dimension. If the patient has not responded to at least half of the questions in the dimension, the score cannot be calculated (missing value).

##### Disability

Disability the DASH (Disabilities of the Arm, Shoulder and Hand measurement tool) (Dubert et al., 2001), is a subjective self-assessment questionnaire for the overall functional capacity of the upper limbs. It evaluates from 30 items the ability to perform 23 activities, the severity of the symptoms and optional sporting or instrumental and professional activity. The entire questionnaire will be administered to the patient. The overall score is in the form of a score on 100 by the following calculation method: [(sum of n responses)−1] / n × 25. A high score indicates a high overall functional disability. The score is valid only if 90% of the questions were filled by the patient (i.e., 3 missing values at most).

##### Fatigue

The MFI 20 (Multidimensional Fatigue Inventory) questionnaire consists of 20 questions divided into four dimensions (Gentile et al., 2003): general and physical fatigue, mental fatigue, the reduction of activities and lack of motivation. Patients will answer questions by circling a number between 1 (“yes it is true”) and 5 (“no, it is not true”) that best applies to what they felt the previous days Administration of the questionnaire. High values indicate a high level of fatigue. This questionnaire appears to be relevant for measuring patient fatigue as it measures the three components of fatigue: physical, psychological and cognitive.

##### Anxiety-Depression

The presence of anxiety and depression symptoms will be measured by the HADS (Hospital Anxiety and Depression Scale) questionnaire (Zigmond and Snaith, 1983). This 14-item scale is used to calculate a standardized anxiety and depression score (0–100).

##### Physical Activity and Sedentary Behavior

Physical activity and sedentary behavior will be measured using the short version of the IPAQ (International Physical Activity Questionnaire) (Craig et al., 2003). This self-administered questionnaire measures the practice of physical activity over the previous 7 days in terms of frequency (number of times per week), duration (number of hours and/or minutes in each session) and intensity (intense, moderate and walking). The combination of these 3 parameters will make it possible to calculate an energy expenditure score related to physical activity in MET-min/week from the (IPAQ Research Committee, 2005). sedentary behavior will be measured by the average time spent sitting per day and according to the context (TV, computer and total time).

#### Qualitative Data

Individual retrospective narrative interviews with 10 patients to question the experience, the difficulties and levers, as well as the adaptations made to the programme, in order to identify the indicators of effectiveness and the necessary adaptations.

### Data Management

#### Data Management Quality Control

Data will be collected and storied in a secure server of the CHRU de Nancy (CIC-1433 EC) using Ennov^®^.

#### Outcomes

The primary outcome is the evolution of QoL score. Secondary outcomes are disability score, fatigue, anxiety-depression, cost-effectiveness and process evaluation.

### Sample Size

The 2 fencing halls have a maximum capacity of 24 patients per year (12 patients per hall). The results of this pilot study will be used to refine the number of subjects needed for the main clinical trial. Nevertheless, with the number of patients to be included, setting an alpha risk of 5%, a power of 80% and assuming normality of the distribution of the QoL score with a standard deviation of 10 points, we will be able to detect a minimum difference in QoL evolution of 8.26 points between the two groups.

### Statistical Analysis

#### Quantitative Analysis

The primary analysis will be performed according to the intention-to-trait principle by comparing the 6-month average evolution of quality of life score between the two groups using mixed model for repeated data. The main criterion of the programme's effectiveness will be assessed by comparing average changes in the 6-month quality of life score between the two groups. An analysis of the missing data will be carried out and the randomness or otherwise of the missing data will be investigated (Diggle and Rubins classification). For the main analysis, a distinction will be made between two types of analysis:

- Univariate analysis allowing the comparison of differences in quality of life scores between the two arms: non-parametric (Mann Whitney-Wilcoxon) and/or parametric (Student Test) methods.
- Multivariate analysis of the repeated customized analysis of variance type taking into account the adjustment and stratification variables (mixed models with consideration of repeated measurements and random center effects). These models make it possible to use not only the measurements at the two extreme times (M0 and M6) but also to take into account the measurement at the intermediate time (M3). They also make it possible to take into account the random center effect (the same intervention will not always have the same effect from one center to another). The SAS MIXED or GLIMMIX procedures will allow this analysis.

Other comparisons will use the same statistical methods for secondary judging criteria.

An analysis of the 6-month change profiles of the different dimensions of quality of life taking into account all monitoring times (0, 3, and 6 months) will be carried out using the Latent Class Growth Analysis (LCGA) methods (Jones et al., 2001; Jung and Wickrama Ka, 2008). This method makes possible to identify sub-groups of patients with distinct trajectories of progression within the entire sample. A comparison of the distribution of these trajectories will be made according to the intervention group.

#### Qualitative Analysis

A thematic analysis of the interviews with patients and masters of arms using NVivo^®^ software will make it possible to describe the appropriateness of the intervention to the realities of the field and the needs of the target population. An automatic speech analysis (detection of verbal recurrences, etc.) will also be carried out using the ALCEST^®^ software.

A cross-check between what the fencing masters say and what the patients say will make it possible to identify the key components of the programme and the areas for improvement. A cross-reference between the patients' discourse and the results on quality of life will be carried out, in an attempt to qualitatively document the evolution. The acceptability of the programme will be evaluated, and the rate of participation/non-participation of patients in each fencing session will be described as a percentage. This will make it possible to estimate the degree of adherence to the intervention.

## Discussion and Perspectives

Regular physical activity (PA) during and after BC treatment has been shown to improve the quality of life (QoL) (Mishra et al., 2012a,b), but studies are still needed to determine the optimal type and timing of exercise (Desnoyers et al., 2016). The RIPOSTE program aimed to propose adapted fencing to patients with BC (Leroy, 2017; Meyer et al., 2017).

Beyond the “*image*” and values conveyed by this sport, many other arguments add to the interest of the practice of fencing. The specific fencing clothing for all and covering the whole body allows to temporarily (i.e., during the adapted fencing sessions) attenuate the visible types of treatments. The guard position of the shoulder of the injured side allows an improvement of the posture that is frequently affected after mastectomy because of an excessive internal rotation of the shoulder after surgery. In addition, the amplitude of the unconscious gesture, educated by the fencing master, pushing the high parries further and further and higher, mobilizes a shoulder and an arm stiffened by scars and surgical adhesions. Laterality, often criticized in this type of sport, becomes an advantage, with patients practicing on the operated side, regardless of their usual laterality. The choice of the weapon is also important because the saber is the lightest weapon in fencing and the targeted areas are above the belt, thus favoring attacks and high parries to the head and therefore needing an adapted shoulder mobilization work. Finally, the elegance of fencing enhances a self-image that is always disturbed after surgery and allows the discovery of a hitherto unknown play activity associated with a consequent level of energy expenditure.

For all these reasons, we believe that adapted fencing according to the scheme of the RIPOSTE program may help patients with BC. We therefore proposed the RIPOSTE pilot randomized controlled trail to challenge the hypothesis that adapted and secure fencing in immediate post-surgery may be an effective way of improving or reducing deterioration of physical and psychological health in patients operated for BC. This would then undoubtedly support and facilitate the widespread of the RIPSOTE program to other fencing club throughout France and abroad.

## Conclusion

Given the specificity of the PA used in this project (fencing), the expected results are not only the improvement of the quality of life of the patients, the reduction of fatigue and anxiety of the patients, but also the improvement of the functional capacities (shoulder) on the operated side. This study will make it possible to envisage possible adaptations of the programme in terms of its content and procedure and will be the basis for the implementation of a larger trial to evaluate the real effectiveness of the Solution RIPOSTE program.

## Strengths and Limitations of This Study

- This is the first study assessing adapted fencing in breast cancer.
- This randomized controlled trial will allow us to precise the most opportune period for initiating a dedicated and adapted fencing program in patients operated for breast cancer.
- Based on strong scientific literature, early adapted fencing may improve physical and psychological health in breast cancer patients.
- A limitation of the study is the small sample size that will limit the impact of the results.

## Ethics Statement

The study protocol has been approved by a French Ethics Committee (CPP Sud Méditerranée IV, N°ID-RCB: 2020-A01916-33).

## Author Contributions

DH-D and MP had the original idea. AO, DP, CR, AV, EA, OH, MT, BC, and MP designed and conceived the protocol. AO, CR, and MP drafted the manuscript. AO, DP, CR, AV, EA, OH, MT, BC, DH-D, and MP critically revised the manuscript for methodology and intellectual content. All authors approved the final version of this manuscript.

## Funding

This study was supported by la Ligue Interrégionale Grand Est contre le Cancer.

## Conflict of Interest

The authors declare that the research was conducted in the absence of any commercial or financial relationships that could be construed as a potential conflict of interest.

## Publisher's Note

All claims expressed in this article are solely those of the authors and do not necessarily represent those of their affiliated organizations, or those of the publisher, the editors and the reviewers. Any product that may be evaluated in this article, or claim that may be made by its manufacturer, is not guaranteed or endorsed by the publisher.
